# Role of asparagine biosynthesis pathway in *Siniperca chuatsi* rhabdovirus proliferation

**DOI:** 10.3389/fmicb.2023.1165491

**Published:** 2023-03-31

**Authors:** Fangying Li, Xiaozhe Fu, Xia Luo, Qiang Lin, Hongru Liang, Yinjie Niu, Lihui Liu, Ningqiu Li

**Affiliations:** ^1^College of Fisheries and Life Sciences, Shanghai Ocean University, Shanghai, China; ^2^Key Laboratory of Fishery Drug Development, Ministry of Agriculture and Rural Affairs, Guangdong Province Key Laboratory of Aquatic Animal Immune Technology, Pearl River Fishery Research Institute, Chinese Academy of Fishery Sciences, Guangzhou, China

**Keywords:** *Siniperca chuatsi* rhabdovirus, asparagine, aspartate metabolism, aspartate–malate shuttle, viral replication

## Abstract

Viruses are non-living organisms that rely on host cellular metabolism to complete their life cycle. *Siniperca chuatsi* rhabdovirus (SCRV) has caused huge economic losses to the Chinese perch (*Siniperca chuatsi*) industry worldwide. SCRV replication is dependent on the cellular glutamine metabolism, while aspartate metabolism plays an important role in viral proliferation in glutamine deficiency. Herein, we investigated roles of asparagine metabolism in SCRV proliferation. Results showed that SCRV infection upregulated the expression of key enzymes in the aspartate metabolic pathway in CPB cells. And the key enzymes of malate-aspartic acid shuttle pathway upregulated during the virus invasion phase, and key enzymes of the asparagine biosynthesis pathway upregulated during the viral replication and release phase. When asparagine was added to the depleted medium, the SCRV copy number restored to 90% of those in replete medium, showing that asparagine and glutamine completely rescue the replication of SCRV. Moreover, inhibition of the aspartate- malate shuttle pathway and knockdown of the expression of key enzymes in the asparagine biosynthesis pathway significantly reduced SCRV production, indicating that the aspartic acid metabolic pathway was required to the replication and proliferation of SCRV. Above results provided references for elucidating pathogenic mechanism of SCRV by regulation of aspartate metabolism.

## 1. Introduction

Replication of viruses completely relies on energy and molecular machinery of host cell (Moreno-Altamirano et al., 2019). Viral infection reconstructs the metabolic pathways of host cells (Thaker et al., 2019), including herpes family (HCMV, HSV-1; Munger et al., 2006; Vastag et al., 2011), dengue virus (DENV; Fontaine et al., 2015), vaccinia virus (VACA; Fontaine et al., 2014), white spot syndrome virus (WSSV; Chen et al., 2011; Su et al., 2014), hepatitis B virus (HBV; Dan Yue et al., 2016; Shi et al., 2016), and adenovirus (Thai et al., 2014, 2015). For example, DENV infection increased glycolysis, intracellular glutamine and glutamate levels, and lipid metabolism (Fontaine et al., 2015; Jordan and Randall, 2017).

*Siniperca chuatsi* rhabdovirus (SCRV) is an important pathogen that seriously harms aquaculture industry and has a high mortality in Chinese perch (*Siniperca chuatsi*; He et al., 1998; Wu et al., 2007). The SCRV genome is a single-stranded negation-strand RNA virus composed of 11,545 nucleotides, encoding only five viral structural proteins (Tao, 2006). Previous study results showed that G protein DNA vaccine can produce higher immune protection against SCRV disease (Chen et al., 2009, 2012). In addition, the N protein was found to be required for replication of SCRV (Zhou et al., 2015). Our previous results indicated that the efficient replication of SCRV is depended on glutamine in CPB cells (Guo Qixi et al., 2019).

Glutamine is the biosynthetic precursor of many molecules, such as amino acids, nucleotides, and fatty acids (Zhang et al., 2017). Glutamine is an important complementation substrate to drive TCA cycle (DeBerardinis et al., 2007). Although *de novo* biosynthesis of some non-essential amino acids is involved in glutamine metabolism, only asparagine biosynthesis is entirely dependent on glutamine (DeBerardinis et al., 2007; Zhang J. et al., 2014). In addition, aspartate synthesis is important in the mitochondrial electron transport chain during cell proliferation processes (Van Vranken and Rutter, 2015). The availability of asparagine is extremely important in biological processes and disease development (Zhang J. et al., 2014). For instance, the contribution of glutamine to cancer cell survival and proliferation is mediated in part by glutamine-dependent asparagine synthesis (Krall et al., 2016). Asparagine restricts VACV protein synthesis through its critical role during VACV replication (Pant et al., 2019). However, it is not clear about the relationship between SCRV replication and aspartate metabolism.

To explore the availability of asparagine in SCRV replication, we investigated expression of enzymes involved in aspartic acid metabolism in SCRV-infected Chinese perch brain cells (CPB). Results indicated that SCRV infection enhanced the expression of major enzymes involved in aspartic acid metabolism. In the absence of glutamine, the addition of asparagine completely rescued SCRV production. Meanwhile, inhibition of malate–aspartate shuttle and knocking down of Asparagine Synthetase (ASNS) significantly downregulated SCRV production in CPB cells. These results provide a new way to study the pathogenesis of SCRV and antiviral treatment strategies.

## 2. Materials and methods

### 2.1. Reagents and antibodies

L-glutamine (Gln, Q), L-asparagine (Asn, N), and D-glucose (Glc) were from Sigma-Aldrich (United States). L-asparagine was dissolved in sterile deionized water (Solarbio) in a water bath at 60°C. Dilute all of the above chemicals to the specified final concentration with the medium prior to use. The rabbit anti-SCRV-N polyclonal antibody was prepared by our laboratory (Fu et al., 2015), and the mouse anti-SCRV-G monoclonal antibody was purchased from Gene create. The rabbit polyclonal antibodies ASNS, GOT1/2, MDH1/2, and mouse anti-β-actin monoclonal antibody were purchased from Proteintech (United States). The goat-anti-mouse and goat-anti-rabbit IgG were purchased from Sigma (United States). Aminooxyacetic acid hemihydrochloride (AOAA) was purchased from MCE (United States), dilution with PBS to the indicated concentration prior to use.

### 2.2. Cell lines and virus strains

The Chinese perch brain cell (CPB) was the sensitive cell lines of SCRV, and the typical cell wire drawing phenomenon occurred in the late stage of SCRV infection (Fu et al., 2015). Chinese perch brain cell (CPB) was established in our laboratory (Fu et al., 2015), which was cultured in Leibovitz L-15 medium (Servicebio, China) supplemented with 10% fetal bovine serum (GIBCO, United States) and maintained at 28°C, the concentration of L-asparagine hydrate in L-15 medium was 1.9 mM, the concentration of L-glutamine was 2.1 mM, and the concentration of D-galactose was 900 mg/L. *Siniperca chuatsi* rhabdovirus (SCRV) was isolated in our laboratory. CPB cells grew to about 90% of the well plate and was infected with SCRV (MOI = 1.0). SCRV was proliferated in CPB cells at 28°C and its titer was determined by TCID_50_ method. Viruses were stored at −80°C until it is used.

### 2.3. Glutamine depletion and rescue

For the study of glutamine deficiency, special DMEM medium from GIBCO, United States, which lacks D-glucose, L-glutamine, sodium pyruvate, and phenol red, was used, and also lacks L-asparagine. The concentrations of all amino acids in the DMEM medium used are detailed in Table 1. The medium was supplemented with 1 g/L D-glucose (Sigma, United States) and 2% dialyzed fetal bovine serum (Hyclone, United States). For glutamine depletion rescue experiments, 1 g/L D-glucose, 2 mM L-glutamine (GIBCO, United States), and 2 mM L-asparagine were added to the medium when necessary. All cells were incubated at 28°C in an incubator with 5% CO_2_. The cells were washed with 1 × phosphate-buffered saline (PBS, VWR) before exposure.

**Table 1:** The concentration of amino acids in DMEM medium.

Essential amino acids	mM
L-Valine	0.4
L-Arginine hydrochloride	0.4
L-Threonine	0.2
L-Histidine hydrochloride-H2O	0.2
L-Isoleucine	0.8
L-Leucine	0.8
L-Lysine hydrochloride	0.8
L-Methionine	0.2
L-Phenylalanine	0.4
L-Tryptophan	0.4
Non-essential amino acids	mM
L-Cystine 2HCl	0.8
L-Serine	0.1
L-Tyrosine disodium salt dihydrate	0.4
Glycine	0.8

### 2.4. Cell viability assay

Cell counting kit-8 (CCK-8) was used to detect the cytotoxicity of glutamine depletion and rescue on CPB cells. Briefly, cells cultured in L-15 medium were seeded in 96-well plate and cultured in an incubator at 28°C. After washing twice with PBS until the cells had grown to about 90%, the cells were fed with 100 μL glutamine-deficient medium, 100 μL glutamine-depleted medium plus 2 mM L-glutamine, or 2 mM L-asparagine, respectively. 72 h after treatment, 10 μL CCK-8 solution was added to each well and incubated at 28°C in a 5% CO2 incubator for 4 h. The viability of OD_450_nm cells was then determined using an ELISA microplate reader (Infinite M200 Pro, Tecan, swiss). Glutamine-free medium was used as a control.

### 2.5. The transcriptional expression of genes was detected by qPCR

Total RNA was extracted from each sample using a cell Total RNA Isolation Kit (FOREGENE, China) and reverse transcription to cDNA was performed according to the protocol of the manufacturer of *TransScript*® All-in-One First-Strand cDNA Synthesis SuperMix for qPCR (One-step gDNA removal) kit (TRANS, China). In order to obtain the cDNA ORF core fragment of ASNS, GOT1/2, and MDH1/2, the primers used are listed in Table 2. Amplification was performed on a real-time PCR instrument using SYBR Green Pro Taq HS kit (AG, China). The reaction volume of SYBR Green was 20 μL, including 10 μL 2 × SYBR Premix Enzyme containing ROX, 1 μL each of forward and reverse primer, and 6 μL ddH_2_O and 2 μL cDNA. All reactions were run three times.

**Table 2:** The primers used in the study.

Primer name	Gene name	Sequence (5 ′ -3 ′)
ASNS-F	Asparagine synthetase	GAAATGTGTGGTATCTGGGCTTTGT
ASNS-R		TCAAAGACTTCAAAGTGGCCAGGG
MDH2-F	Malate dehydrogenase 2	ACTGCCCCGAAGCTATGA
MDH2-R		CAGCGTGACCTCCAATGAC
GOT2-F	Glutamic-oxaloacetic transaminase	ACCATGGCCCTGCTCAAG
GOT2-R		TACTTGGTGACAGCATGGATCGC
GOT1-F	Glutamic-oxaloacetic transaminase 1	CGGCTTATCAGGGTTTCGC
GOT1-R		CAGGGCTGTTGAGGGTCTTG
MDH1-F	Malate dehydrogenase 1	ATGGGTGTTTACTCCTCTGGC
MDH1-R		TTTGGGGCGGTCTAAGTG
18S-F	18S RNA	CATTCGTATTGTGCCGCTAGA
18S-R		CAAATGCTTTCGCTTTGGTC
SCRV-F	SCRV-Rhabdovirus 140	GACATGTTCTTCTACAGATTCAAC
SCRV-R		CAATCCAGCACTCCACTG
SCRV-Probe		AGGTTCAAAGACTGTGCAGCTCTGT

### 2.6. RNA extraction and quantification of SCRV genome copies

Total RNA from intracellular and supernatant of SCRV-infected CPB cells were extracted with Easy Pure Simple viral DNA/RNA kit (Trans Gen Biotech, China). Then, after detecting the concentration of RNA samples and reverse transcription to cDNA was performed according to the protocol of the manufacturer of *TransScript*® One-step gDNA removal kit (TRANS, China), the ORF 140 gene of SCRV was quantified by Pro Taq HS Premix Probe qPCR Kit. The reaction system is: containing 2 × Premix 10 μL, 0.5 μL of each primer (10 μM), 0.5 μL Probe, 0.5 μL ROX, and 6 μL ddH_2_O. The procedure was: 94°C for 1 min, 94°C for 10 s, and 60°C for 30 s, 40 cycles. There are three biological replicates for each target gene. The used primers and probe were listed in Table 2.

### 2.7. Western blot

Chinese perch brain cells were collected, and total proteins were extracted by RIPA and 1 mM PMSF lysis. After boiling for 10 min, SDS-PAGE was performed. The 20× rapid membrane transfer buffer (Yoche, Shanghai) was diluted into 1× working concentration in advance, and then the filter paper, glue, PVDF membrane, and filter paper were placed in the sequence before the film transfer operation was carried out on the film transfer instrument. Then, the converted PVDF membrane was placed in PBST containing 5% (w/v) skim milk powder for 3 h at room temperature. Then primary antibodies ASNS (1:400), MDH2 (1:1,000), GOT2 (1:500), GOT1 (1:500), MDH1 (1:500), SCRV-N (1:1,000), SCRV-G (1:1,000), and β-actin (1:3,000) were diluted according to the instructions, placed on shaking table for 3 h or 4°C overnight incubation. The primary antibody was discarded, rinsed with PBST for three times, and then the secondary antibody was diluted at a certain ratio (1:5,000), and incubated for 1 h at room temperature with shaking. The bands were colored using HRP ECL (Millipore, United States) or DAB color solution (TIANGEN, China).

### 2.8. RNA interference

The siRNA sequence of ASNS was designed by Shanghai Genopharmaceutics Co., Ltd. based on the sequence of ASNS (NCBI Reference Sequence: XM-044171730.1; Table 3). si-ASNS and NC were transfected into CPB cells grown to 70–80% by CPB using TransIntro™ EL transfection reagent (Trans-Gen Biotech), and cell-like and protein-like were collected at 24 and 72 h after transfection, respectively. The negative control (NC) group was used as the control group and qPCR and western blotting were performed to determine the siRNA sequence that best knocked down ASNS. Then, the siRNA and NC with the best knockdown effect were transfected into CPB cells, and the cells were inoculated 4 h later. At 72 h, the cell samples and proteins were collected for qPCR and WB detection to analyze the effect of ASNS knockdown on SCRV virus production and viral protein.

**Table 3:** Interfering RNA sequence of ASNS.

Name	Sequence (5′–3′)	Anti-Sequence (5′–3′)
siRNA-ASNS (siASNS-1)	GGCUUUGUUUGGCAGUGAUTT	AUCACUGCCAAACAAAGCCTT
siRNA-ASNS (siASNS-2)	GCAAGGUUGCAGCUCACAUTT	AUGUGAGCUGCAACCUUGCTT
siRNA-ASNS (siASNS-3)	GGUAUGUACUUGGUUUCAATT	UUGAAACCAAGUACAUACCTT
Negative control (NC)	UUCUCCGAACGUGUCACGUTT	ACGUGACACGUUCGGAGAATT

### 2.9. Statistical analysis

GraphPad Prime 5 software was used for data collation and graphing. The analysis was performed using one-way ANOVA or two-way ANOVA, and all data were obtained from three parallel experiments. The notational convention for statistical significance is as follows: NS, *p* > 0.05; ^*^p ≤ 0.05; ^**^p ≤ 0.01; and ^***^p ≤ 0.001.

## 3. Results

### 3.1. SCRV infection altered aspartic acid metabolism

**Figure 1:**
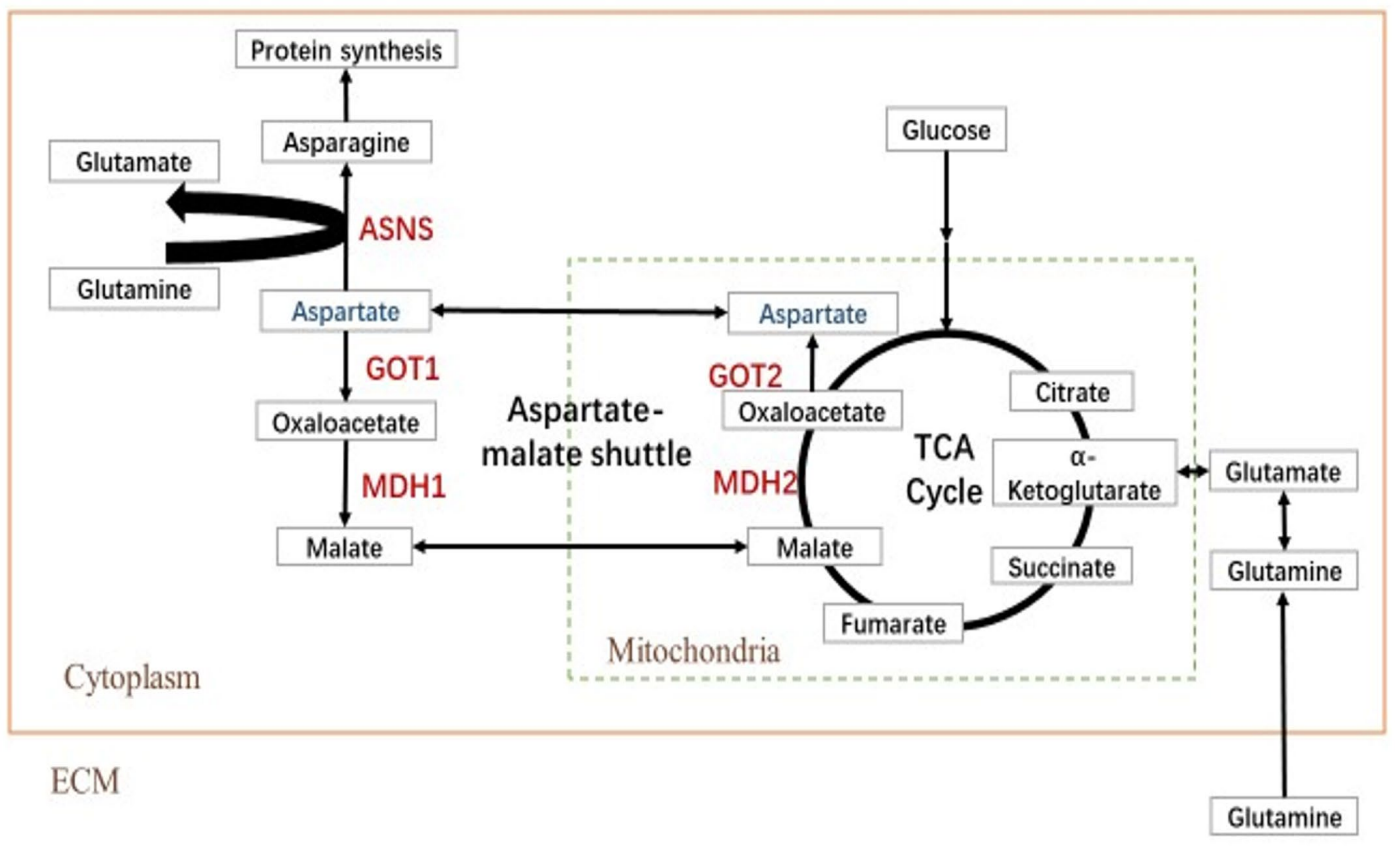
Simplified schematic of aspartate metabolism. Reactions within the box are intracellular, and inside the dotted box occurs in the mitochondrion (Mito), whereas those outside in the dotted box occur in the cytoplasm (Cyto). TCA cycle, tricarboxylic acid cycle; MDH, malate dehydrogenase; GOT, glutamic oxalacetate transaminase; and ASNS, asparagine synthetase.

**Figure 2:**
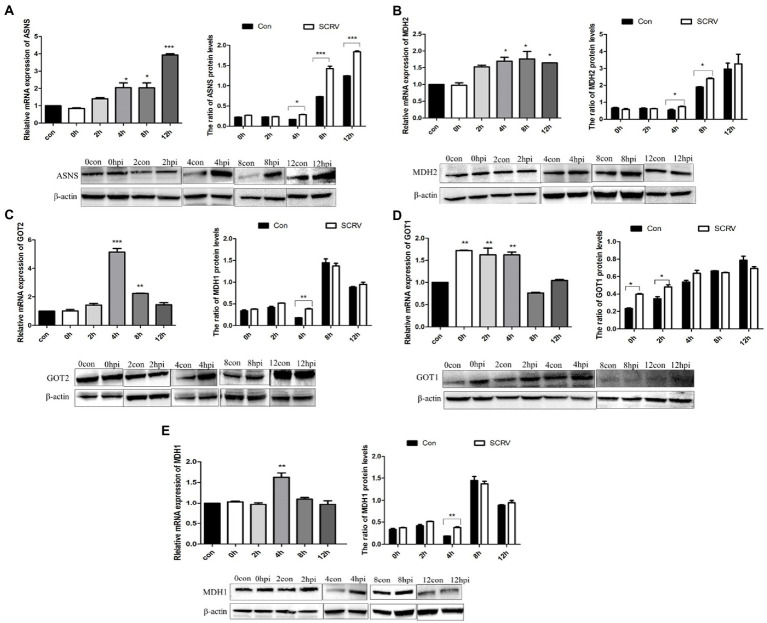
SCRV infection altered aspartate metabolism in CPB cells. Cells infected with or without SCRV (MOI = 1.0) were collected at 12, 24, 36, 48, and 72 h for transcript and protein levels analysis. The transcript levels of key enzymes of the aspartate metabolic pathway were compared with the uninfected group. **(A)** Transcription level and protein level of ASNS, the key enzyme in asparagine biosynthesis pathway (*n* = 3). **(B–E)** Transcript levels and protein levels of the key enzyme genes MDH2, GOT2, GOT1, and MDH1 in the aspartic acid-malate shuttle pathway. ASNS, asparagine synthetase; MDH1/2, malate dehydrogenase 1/2; GOT1/2, glutamic oxalacetate transaminase 1/2; con, control; and hpi, hour post infection. NS, *p* > 0.05, ^*^p < 0.05, ^**^p < 0.01, ^***^p < 0.001 (unpaired Student’s *t*-test). Data are representative of three different independent experiments (mean ± SEM).

The transfer of aspartic acid to mitochondria and its conversion to oxaloacetic acid is sufficient to drive the TCA cycle to produce energy and other biomolecules (Dornfeld et al., 2015). The key metabolites of the aspartic acid metabolic pathway are shown in Figure 1. To investigate the role of aspartic acid metabolism in SCRV proliferation, the expression of ASNS, MDH2, GOT2, GOT1, and MDH1 in mRNA level and protein level were detected in CPB cells at different time points by qPCR and western blot. Among them, MDH1/2 and GOT1/2 are the key enzymes of malate–aspartate shuttle (MAS) pathway, and ASNS is the key enzyme of asparagine biosynthesis pathway. The medium used was L-15 medium (Servicebio, China) containing 900 mg/L of D-galactose, 1.9 mM of L asparagine-hydrate, and 2.1 mM of L-glutamine. As seen in Figure 2A, the expression of ASNS was increased during 4–12 hpi in mRNA level and protein level. For key enzymes in the malate-aspartic acid shuttle pathway, the expression of MDH2 and GOT2 was significantly increased during 4–8 hpi in mRNA level and protein level (Figures 2B,C). For the GOT1 and MDH1, the expression of GOT1 was upregulated during 0–2 hpi (Figure 2D), and expression of MDH1 was upregulated at 4 hpi (Figure 2E). Above results indicated that SCRV infection mainly promoted the expression of GOT1 in the early stage, and promoted the expression of MDH2, GOT2, and ASNS in CPB cells in the late stage. Our data suggested that SCRV infection leads to remodeling of aspartate metabolism in CPB cells. The malate–aspartate shuttle pathway is significantly promoted in the early stage of SCRV replication, which promotes the synthesis and shuttle of aspartate and provides the raw material for asparagine synthesis. In the late stage of SCRV replication, SCRV infection significantly enhanced the asparagine biosynthesis pathway, promoting the synthesis of asparagine from aspartate for virus production.

### 3.2. Asparagine rescues SCRV replication in glutamine depletion

**Figure 3:**
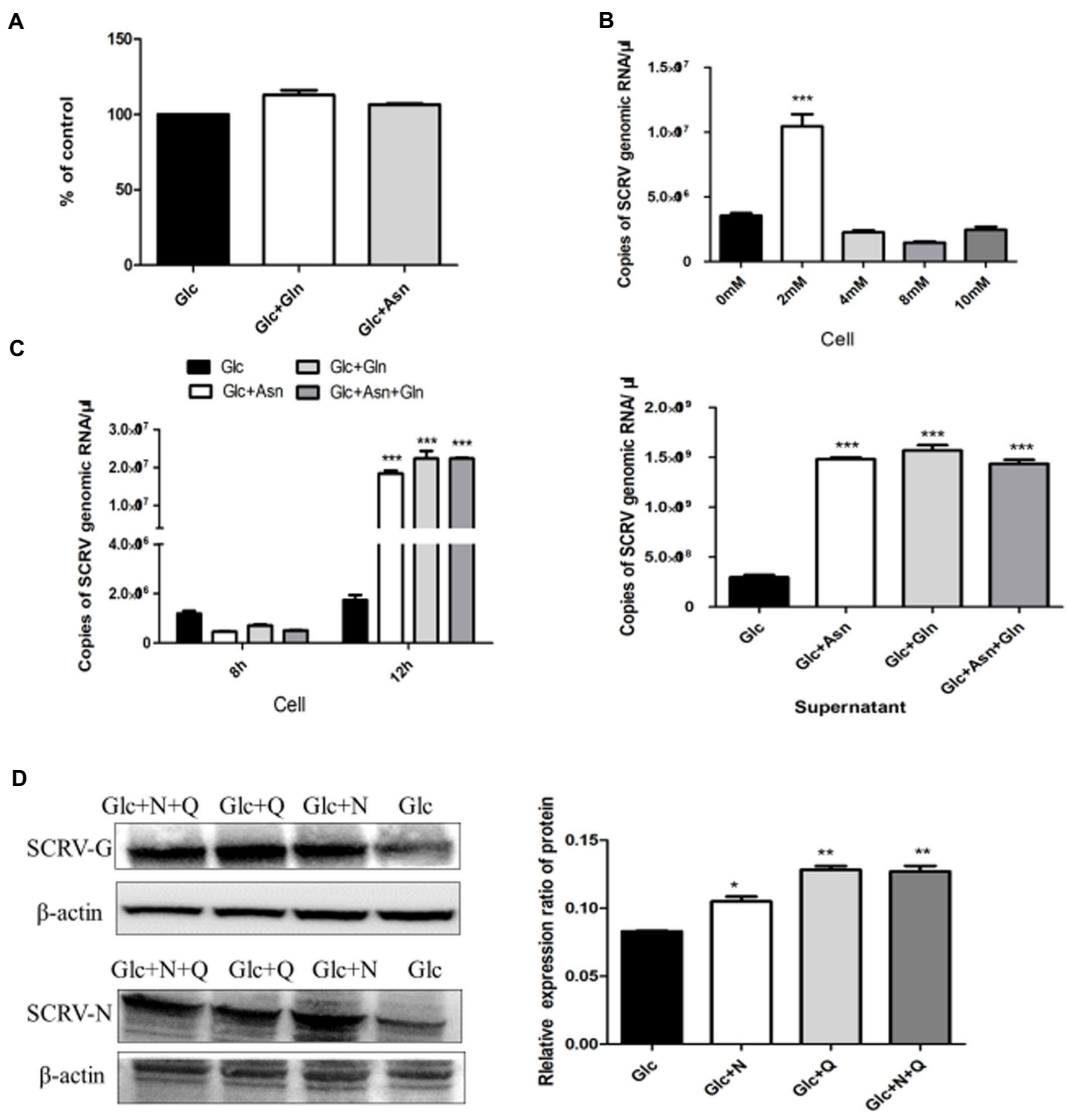
Asparagine recused SCRV replication in glutamine depletion. **(A)** Cell viability of CPB. Cells were fed with medium containing only 1 g/L glucose (Glc), 1 g/L glucose plus 2 mM glutamine (Glc + Gln), and 1 g/L glucose plus 2 mM asparagine (Glc + Asn). At 72 h post treatment, cell viability was measured by CCK8 assay. **(B)** Optimal rescue concentration of asparagine. The number of virus copies in the medium containing glucose plus 0, 2, 4, 8, and 10 mM asparagine were determined by qPCR, respectively. **(C)** Comparison of virus production. CPB cells were infected with SCRV at an MOI of 1 and fed media containing 1 g/L glucose (Glc), 1 g/L glucose plus 2 mM asparagine (Glc + Asn), 1 g/L glucose plus 2 mM glutamine (Glc + Gln), and 1 g/L glucose plus 2 mM asparagine plus 2 mM glutamine (Glc + Asn + Gln) at 1 hpi. Intracellular virus was harvested at 8 hpi, and virus in the cells and supernatant was harvested at 12 hpi. **(D)** Western blot was used to detect and analyze the expression levels of SCRV G and N proteins in SCRV infected cells. CPB cells were infected with SCRV at an MOI of 1 and were given1g/L glucose (Glc), 1 g/L glucose plus 2 mM asparagine (Glc + N), 1 g/L glucose plus 2 mM glutamine (Glc + Q), and 1 g/L glucose plus 2 mM asparagine plus 2 Mm glutamine (Glc + N + Q) media at 1 hpi. Proteins in the cells were harvested at 12 hpi (*n* = 3). The control group consisted of glucose only. ^*^p ≤ 0.05, ^**^p ≤ 0.01, ^***^p ≤ 0.001. Data are representative of three different independent experiments (mean ± SEM).

Previous results showed that glutamine loss for 72 h had no obvious effect on CPB cell viability (Fu et al., 2017). The CPB cells were cultured in glutamine-free DMEM medium for 72 h, compared with control cells with only glucose, and the 72-h proliferation rate with the addition of asparagine was 106% (Figure 3A), indicating that asparagine had no effect on cell viability within 72 h of CPB. To determine the optimal supplemental concentration of asparagine to rescue the SCRV titer, virus production with different supplemental concentrations of asparagine was measured by qPCR. As seen in Figure 3B, the addition of 2 mM asparagine significantly increased the replication of SCRV.

To determine the phase requirements of glutamine and asparagine in the SCRV life cycle, we analyzed viral production and viral protein synthesis in CPB cells cultured in medium with or without glutamine and asparagine by qPCR and WB assays. Previous studies showed that the replication and proliferation cycle of SCRV in CPB cells was 12 h, and the growth rate was significantly accelerated at 8–12 h (Thaker et al., 2019). As shown in Figure 3C, compared with the control group, there was no significant change in intracellular virus production in the presence of glutamine or asparagine at 8 h post infection, but at 12 h post infection, intracellular SCRV production was significantly upregulated when in the presence of glutamine or asparagine. Furthermore, in the supernatant SCRV yield was also increased at 12 h post infection when in the presence of glutamine or asparagine, compared to the control group. Notably, intracellular and extracellular viral production in the medium obtaining glutamine and asparagine were no difference to those only obtaining glutamine at 8 and 12 h post infection. These results indicated that SCRV replication proliferation could be rescued by asparagine when glutamine deletion. Next, we examined expression of the SCRV N/G proteins. As shown in Figure 3D, N and G protein synthesis was promoted when the SCRV infected cells were supplemented with asparagine in glutamine-free medium, suggesting that asparagine rescues glutamine-deficient SCRV protein synthesis. Taken together, these experiments showed that asparagine could completely rescue the SCRV production in the absence of glutamine.

### 3.3. Inhibition of the aspartate-malate shuttle affects SCRV replication

**Figure 4:**
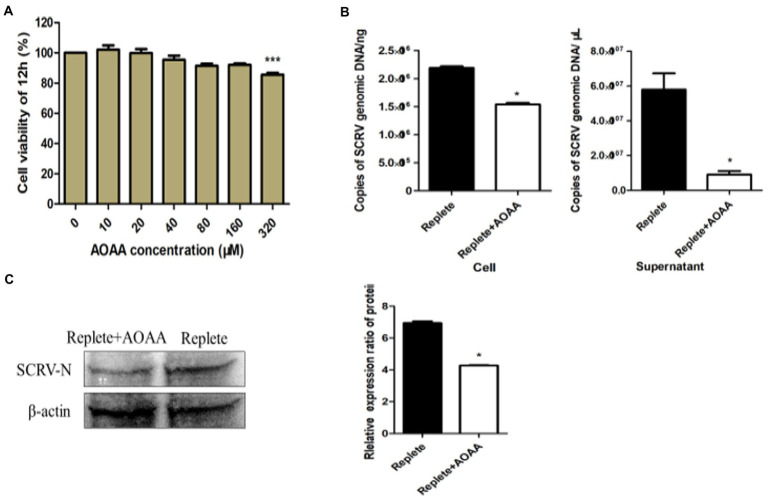
Inhibition of the aspartate-malate shuttle affects SCRV replication. **(A)** Cell viability with added inhibitors. Different concentrations of aminooxyacetic acid hemihydrochloride (AOAA) were added to glutamine medium, and after 12 h of treatment, cell viability was determined by CCK-8 assay. The activity of mock-treated samples was assigned as 100%. **(B)** Effect of addition of inhibitor AOAA on SCRV virus yield. CPB cells were infected with SCRV at an MOI of 1 in the presence or absence of AOAA (100 μmol) in glutamine medium, intracellular and supernatant SCRV were harvested at 12 hpi, and virus production was determined by qPCR. **(C)** Expression of SCRV viral proteins by adding inhibitor AOAA. CPB cells were infected with SCRV at an MOI of 1 in the presence or absence of AOAA (100 μmol) in glutamine medium, and intracellular proteins were collected at 12 hpi, and the expression of SCRV-N protein was detected by western blot analysis. NS, *p* ≥ 0.05, ^*^p ≤ 0.05, ^**^p ≤ 0.01, ^***^p ≤ 0.001. (unpaired Student’s *t*-test). Data are representative of three different independent experiments (mean ± SEM).

Some researchers have found that MAS has a great relationship with the biosynthesis of glutamate (Dienel and Cruz, 2016) and glutamine (Madhu et al., 2016). Some studies have shown that GOT plays an irreplaceable role in the shuttle system driven by aspartic acid-glutamate carrier (Fitzpatrick et al., 1983). AOAA is a specific inhibitor of GOT, and we inhibited the MAS process by adding AOAA. CCK-8 method was used to detect the effect of addition of 0–320 μmol AOAA on the viability of CPB cells. As shown in Figure 4A, the addition of 160 μmol AOAA had no significant effect on the viability of CPB cells. When 100 μmol AOAA was added to the complete medium, the results showed that the production of SCRV virus was significantly downregulated, decreasing by 49.3% in intracellular and 23.7% in supernatant (Figure 4B). The effect of AOAA on SCRV protein expression was further detected by Western blot. As shown in Figure 4C, the protein expression of the N protein gene of SCRV was significantly downregulated. In conclusion, inhibition of the aspartic acid-malate shuttle would inhibit the replication and proliferation of SCRV.

### 3.4. ASNS knockdown impairs SCRV replication

**Figure 5:**
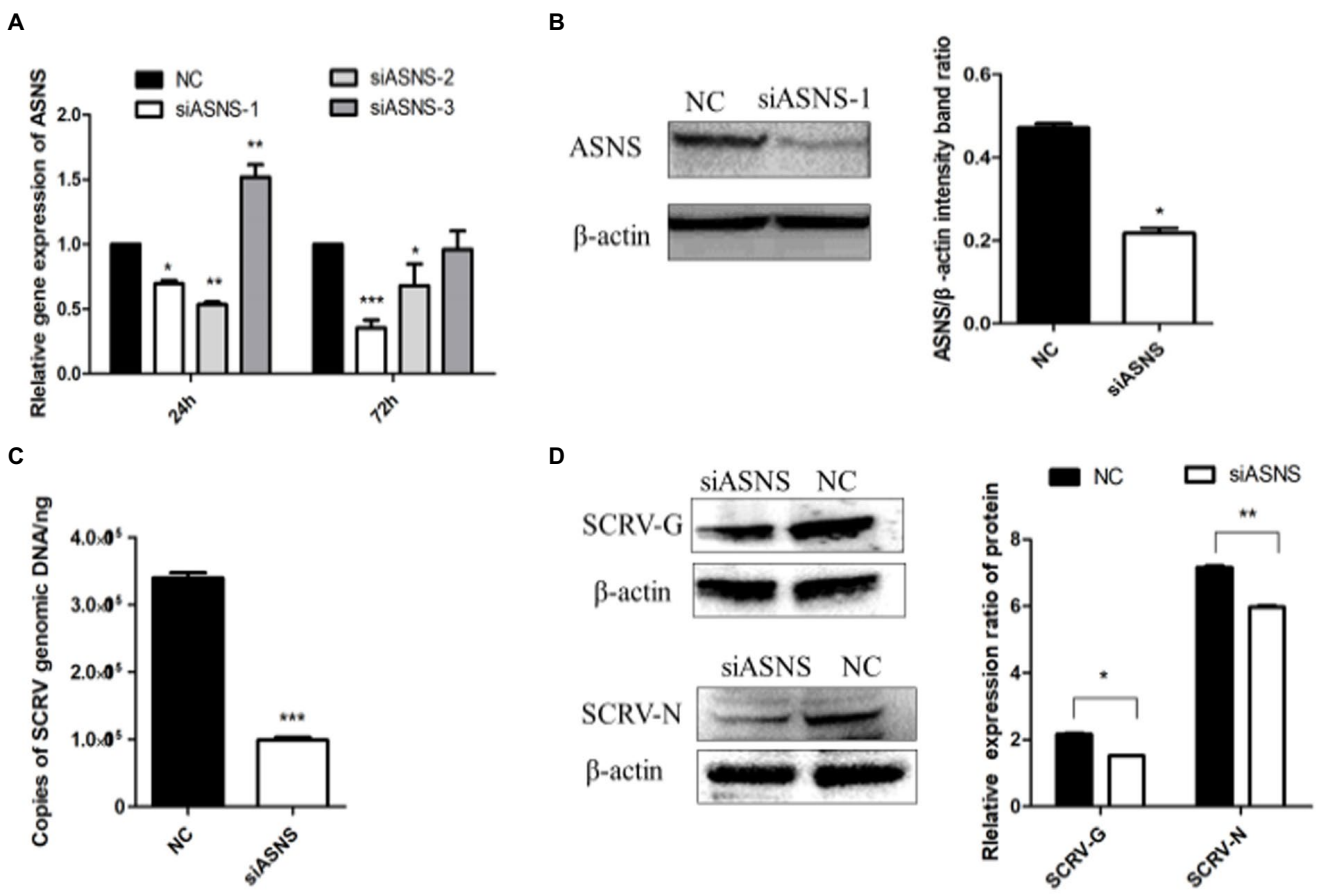
ASNS knockdown impaired SCRV replication. **(A)** The expression of ASNS mRNA in CPB cells treated with si-ASNS was detected. CPB cells fed with glutamine-containing medium were transfected with the three siRNAs and negative control (NC), and the cells were harvested at 24 and 72 h, respectively, and the transcript level of ASNS was detected by qPCR. **(B)** Validation of siRNA. The protein in the cells was harvested 72 h after siASNS-1 transfection, and western blot was used to verify whether siASNS-1 effectively knocked down the protein expression of ASNS. **(C)** The yield of SCRV when ASNS is knocked down. The cells were infected with SCRV (MOI = 1.O) 24 h after siASNS-1 transfection. The cells were collected at 72 hpi and the virus production was measured by qPCR. **(D)** Expression of SCRV-G/N protein in CPB cells after ASNS knockdown. Cells were infected with SCRV (MOI = 1.O) 24 h after siASNS-1 transfection. The protein in the cells was collected at 72 hpi, and the protein expression was detected by western blot. ^*^p ≤ 0.05, ^**^p ≤ 0.01, ^***^p ≤ 0.001. (unpaired Student’s *t*-test; *n* = 3). Data are representative of three different independent experiments (mean ± SEM).

The *de novo* synthesis of asparagine uses glutamine to provide amino groups and is catalyzed by asparagine synthetase. To test whether the biosynthetic pathway of asparagine affects SCRV replication, small RNAs interfering with ASNS were designed and synthesized. As shown in Figures 5A,B compared to NC group, expression of ASNS were significantly decreased in the mRNA and protein level at 72 h post siASNS-1 treatment, indicating that siASNS-1 can knockdown expression of ASNS. To explore the effect of ASNS knockdown on SCRV replication, CPB cells were first treated with si-ASNS and then infected with SCRV at 24 h. The results showed that the copy number of SCRV in CPB cells was decreased by 60.7% by knockdown of ASNS (Figure 5C). Knockdown of ASNS significantly impaired SCRV replication. In ASNS siRNA-treated cells, the SCRV-G/N protein synthesis was downregulated (Figure 5D). These results indicated a critical role of asparagine biosynthesis in SCRV replication.

## 4. Discussion

The SCRV infection caused serious economic losses to the Chinese perch farming industry in China. It has been shown that aspartate metabolism is regulated in mammalian cells (Pant and Yang, 2020). Many cancer cells rely on increased aspartate metabolism to proliferation. As well as cancer cells, viruses required increased anabolism to support proliferation (Krall et al., 2016). In this study, we found that CPB cells infected with SCRV promoted the expression of enzymes related to aspartic acid metabolism. Results indicated that SCRV infection mainly promoted the expression of GOT1 in the early stage and promoted the expression of MDH2, GOT2, and ASNS in CPB cells in the late stage, illuminating that SCRV infection drives the malate-aspartic acid shuttle and the biosynthesis of asparagine. Previous studies showed that ASNS play an important role in the replication and proliferation of HCMV and VACA viruses (Van Vranken and Rutter, 2015; Pant et al., 2019). Moreover, adenovirus infection upregulated expression of ASNS in infected cells, whereas knockdown of ASNS severely impaired viral replication (Stagliano et al., 2003; Thaker et al., 2019). Our results were consistent with those of adenovirus. These results suggested that some viruses have evolved ways to regulate asparagine biosynthesis. Both MDH1/2 and GOT1/2 are highly expressed in the malate-aspartic acid shuttle pathway of lung adenocarcinoma, and the high expression of GOT2 may promote the occurrence and malignant progression of lung adenocarcinoma (Xin Xiang-bing et al., 2019). Our results confirmed that SCRV infection upregulated the expression of malate-aspartic acid shuttle MDH1/2 and GOT1/2. In a previous study, it was found that the use of MAS inhibitor AOAA mainly inhibited the activity of GOT (Lee et al., 2011). Our results showed that the addition of AOAA inhibited the production of SCRV virus and the expression of viral proteins, but the mechanism of its action in virus replication and proliferation needs further study.

Proteins and amino acids play an important role in maintaining body structure and metabolism. The protein requirement of fish is actually a requirement for the mixing ratio of essential and non-essential amino acids (Lee et al., 2019). DMEM medium contains essential amino acids for fish growth to maintain basal cell metabolism (Table 1), these amino acids can be broken down into TCA cycle intermediates into the TCA cycle, but then will not be converted into asparagine and glutamine (De and Zhang, 2018). In addition, glutamine provides carbon and nitrogen atoms to synthesize nucleotides and other NEAAs. It is important to note that carbon and nitrogen atoms can be obtained directly from glutamine or glutamine-derived metabolites in multiple biosynthetic steps. The nitrogen atom in aspartic acid is synthesized from glutamic acid by transamination (Jiang et al., 2019). During viral infection, glutamine metabolism increases, which compensates for glucose consumption. Moreover, glutamine is also required for ASNS catalysis in asparagine biosynthesis (DeBerardinis et al., 2007; Zhang J. et al., 2014). Several recent studies have shown that asparagine can limit the replication of VACA and human cytomegalovirus (HCMV; Gao et al., 2012). Our results show that the addition of 2 mM asparagine is the optimal concentration to rescue SCRV replication, while high concentrations of asparagine inhibit cell growth (Zhang W. Y. et al., 2014). Our findings suggested that in the absence of glutamine, asparagine rescued viral production in the later stages of SCRV infection of CPB cells. Possibly providing a new source of nitrogen for cell growth. This result is consistent with those of VACV. Lee et al. also reported the importance of asparagine metabolism during the acute replication of HCMV (Gao et al., 2012). HCMV replication is significantly restricted when ASNS is knocked down. These experiments were performed in the presence of glutamine, so our results suggest that asparagine biosynthesis plays a key role in SCRV replication. In fact, in physiological conditions where protein synthesis is in high demand, asparagine supply could also be a limiting factor. Consistent with these observations, there is growing evidence that asparagine is essential in controlling metabolic availability in biological processes. When cells are deficient in several different amino acids, they enhance asparagine biosynthesis to regulate gene expression (Gong et al., 1991; Hutson and Kilberg, 1994). These results can lay a foundation for elucidating the molecular mechanism of SCRV regulating the metabolism of aspartic acid in host cells, and provide a new idea for elucidating the pathogenic mechanism of SCRV and antiviral treatment strategy.

## 5. Conclusion

In summary, SCRV infection hijacked a variety of enzymes to accelerate the aspartic acid metabolic pathway in CPB cells, and provided energy and biomolecules for viral replication and proliferation. Replication of SCRV is highly dependent on levels of the asparagine, and inhibition of a key enzyme involved in asparagine synthesis results in a severe weakening of viral replication. These findings suggest that the key enzymes of aspartic acid metabolism pathway may be a new target to effectively control SCRV infection, providing a new idea for the treatment strategy of SCRV disease.

## Data availability statement

The raw data supporting the conclusions of this article will be made available by the authors, without undue reservation.

## Author contributions

NL: conceptualization, funding acquisition, and supervision. FL, XF, XL, QL, HL, YN, and LL: investigation. FL: writing—original draft. All authors contributed to the article and approved the submitted version.

## Funding

This study was supported by National Natural Science Fund (32273181), Special Funds for Marine Economy Development (Six marine industries) of Guangdong Province [GDOE(2022)A13], Guangdong Basic and Applied Basic Research Foundation (2020B1515120012), Science and Technology Program of Guangzhou (202102080275), Central Public-interest Scientific Institution Basal Research Fund, CAFS (NO.2019SJ-XT4), and Guangdong Provincial Special Fund for Modern Agriculture Industry Technology Innovation Teams (2019KJ140 and 2019KJ141).

## Conflict of interest

The authors declare that the research was conducted in the absence of any commercial or financial relationships that could be construed as a potential conflict of interest.

## Publisher’s note

All claims expressed in this article are solely those of the authors and do not necessarily represent those of their affiliated organizations, or those of the publisher, the editors and the reviewers. Any product that may be evaluated in this article, or claim that may be made by its manufacturer, is not guaranteed or endorsed by the publisher.
